# Genes for adaptation and learning spanning evolution: computational comparison between synaptic transmission and chemo-tactic signaling protein networks

**DOI:** 10.1186/1471-2202-12-S1-P97

**Published:** 2011-07-18

**Authors:** Riham Satti, Gillian Deakin, Reiko J Tanaka, Aldo Faisal

**Affiliations:** 1 Department of Bioengineering, Imperial College London, London, SW7 2AZ, UK; 2 Institute for Mathematical Sciences, Imperial College London, London, SW7 2AZ, UK; 3 Department of Computing, Imperial College London, London, SW7 2AZ, UK

## 

Plasticity at the synapse and adaptation in bacterial chemotaxis are two prominent examples of biological regulation and signal processing in response to noisy, time-varying stimuli [1,2]. Both regulatory systems process glutamate stimuli (as neurotransmitter and food respectively) to determine whole-cell response to future changes in glutamate concentration. These two evolutionary distant protein networks thus perform a common computational function (adaptation to stimulus patterns) on glutamatergic inputs. Moreover, the bacterial glutamate receptor is an evolutionary ancestor of mGlu and NMDA receptors in the mammalian synapse [1,3]. Thus, we were curious if common regulatory principles of both networks and specifically if their proteins had common evolutionary roots. We investigated this hypothesis by performing a comparative bioinformatics study to test if the amino acid sequences of these two protein networks are conserved across 600 Million years.

We focussed on mouse (*M. Musculus*) post-synaptic proteins [4] and the 23 proteins involved in bacterial chemotaxis of *E.coli*[2,5], both available on the UniProt protein database. We measured protein similarity by aligning the sequences of all synaptic *M.Musculus* proteins (tagged as “synapse” related in UniProt) with all 23 bacterial chemotaxis proteins, using the local pairwise Smith-Waterman algorithm [6]. Because the algorithm’s similarity score is sequence length dependent and evolutionary distance between proteins results in considerable genetic drift, the comparison is difficult [7]. Therefore, we developed a normalization method to establish significance of alignments (cf. Figure): We established two baseline sets of alignments: we aligned 300 generic (non-synpatic) proteins from *M.Musculus* (with similar length as the synaptic proteins) with the 23 bacterial chemotaxis proteins and vice versa 100 generic (non chemo-tactic) *E.coli* proteins (of similar length as the chemo-tactic proteins) with the 84 synpatic proteins from *M.Musculus.* Any significant sequence similarity score between synaptic and chemotactic proteins would have to stands out from the large set of scores of the baseline sets: We normalised the alignment score S_ij_, between a synaptic protein *i*, and a chemo-tactic protein *j*, using the mean μ_ij_, and standard deviation σ_ij_ of the baseline score distributions. Thus, alignments between synaptic and chemo-tactic proteins with positive normalized scores, indicated strong sequence similarity.

We found a set of a dozen synaptic and chemotactic proteins that show high sequence similarity across this vast evolutionary gap. The highest scoring one was, for example, the methyl-accepting chemotaxis protein III in bacteria and the glutamate receptor interacting 1 (GRIP1) associated protein in synaptic transmission. GRIP1 is an adapter protein linking AMPA receptors associated to increase synaptic efficiency [8], whereas methyl-accepting chemotaxis protein III is involved in adaptation by varying the level of methylation to allow bacteria to remain sensitive to changes in average glutamate concentration [9]. This novel link suggests regulatory networks for adaptation and learning at the synapse have common ancestors and possible common principles (see also [10]). We pose the idea, that like Mitochondria (which were bacteria integrated into eukaryotic cells to supply energy) so could ‘learning’ at the chemical synpase be the result of integrating the chemotaxis network into early neurons.
